# A Naturally-Derived Compound Schisandrin B Enhanced Light Sensation in the *pde6c* Zebrafish Model of Retinal Degeneration

**DOI:** 10.1371/journal.pone.0149663

**Published:** 2016-03-01

**Authors:** Liyun Zhang, Lue Xiang, Yiwen Liu, Prahatha Venkatraman, Leelyn Chong, Jin Cho, Sylvia Bonilla, Zi-Bing Jin, Chi Pui Pang, Kam Ming Ko, Ping Ma, Mingzhi Zhang, Yuk Fai Leung

**Affiliations:** 1 Department of Biological Sciences, Purdue University, 915 W. State Street, West Lafayette, IN, 47907, United States of America; 2 Laboratory for Stem Cell & Retinal Regeneration, Division of Ophthalmic Genetics, The Eye Hospital of Wenzhou Medical University, Wenzhou 325027, China; 3 Department of Statistics, University of Georgia, 101 Cedar St, Athens, GA 30602, United States of America; 4 Department of Ophthalmology and Visual Sciences, Chinese University of Hong Kong, Hong Kong, China; 5 Division of Life Science, The Hong Kong University of Science & Technology, Clear Water Bay, Hong Kong, China; 6 Joint Shantou International Eye Center, Shantou University & the Chinese University of Hong Kong, Shantou, China; 7 Department of Biochemistry and Molecular Biology, Indiana University School of Medicine Lafayette, 625 Harrison Street, West Lafayette, IN 47907, United States of America; University of Massachusetts Medical School, UNITED STATES

## Abstract

Retinal degeneration is often progressive. This feature has provided a therapeutic window for intervention that may extend functional vision in patients. Even though this approach is feasible, few promising drug candidates are available. The scarcity of new drugs has motivated research to discover novel compounds through different sources. One such example is Schisandrin B (SchB), an active component isolated from the five-flavor fruit (*Fructus Schisandrae*) that is postulated in traditional Chinese medicines to exert prophylactic visual benefit. This SchB benefit was investigated in this study in *pde6c*^*w59*^, a zebrafish retinal-degeneration model. In this model, the *pde6c* gene (*phosphodiesterase 6C*, *cGMP-specific*, *cone*, *alpha prime*) carried a mutation which caused cone degeneration. This altered the local environment and caused the bystander rods to degenerate too. To test SchB on the *pde6c*^*w59*^ mutants, a treatment concentration was first determined that would not cause morphological defects, and would initiate known physiological response. Then, the mutants were treated with the optimized SchB concentration before the appearance of retinal degeneration at 3 days postfertilization (dpf). The light sensation of animals was evaluated at 6 dpf by the visual motor response (VMR), a visual startle that could be initiated by drastic light onset and offset. The results show that the VMR of *pde6c*^*w59*^ mutants towards light onset was enhanced by the SchB treatment, and that the initial phase of the enhancement was primarily mediated through the mutants’ eyes. Further immunostaining analysis indicates that the treatment specifically reduced the size of the abnormally large rods. These observations implicate an interesting hypothesis: that the morphologically-improved rods drive the observed VMR enhancement. Together, these investigations have identified a possible visual benefit of SchB on retinal degeneration, a benefit that can potentially be further developed to extend functional vision in patients.

## Introduction

Retinal degeneration is a group of retinal disorders that are incurable [1,2]. These disorders affect photoreceptors (PRs), the light-sensitive neurons in the retina. There are two types of PRs: rods for dim-light vision, and cones for bright-light and colour vision. These PRs degenerate due to either intrinsic genetic mutations or as a consequence of the changing local cellular environment. The resulting PR loss would permanently impair the patients’ vision. Nonetheless, the retinal-degeneration process is often progressive so that the affected PRs die gradually [3]. This feature has provided a therapeutic window through which drugs can be applied to slow disease pathogenesis and extend patients’ functional vision.

The feasibility of this drug-treatment approach has recently been demonstrated by two studies. One of them tested an antioxidant mixture of α-tocopherol, ascorbic acid, Mn(III) tetrakis porphyrin, and α-lipoic acid on the *retinal degeneration 1* (*rd1*) mouse [4]. This mutant had a homozygous nonsense mutation in the rod-specific *Pde6b* gene, causing severe early-onset retinitis pigmentosa (RP). In the mutant, rods died due to the mutation, while cones died amidst the changing local environment caused by rod death. When the *rd1* mutant was treated with the drug mixture, cone death was reduced and cone function was preserved. In a subsequent study, the same mixture was tested on two additional models: the *retinal degeneration 10* (*rd10*) mouse and the *Q344ter* mouse [5]. The *rd10* mice carried a homozygous missense mutation in the *Pde6b* gene, which resulted in recessive RP that was slowly progressive; whereas the *Q344ter* mice carried a truncation in the *Rho* gene, causing dominant RP that was rapidly progressive. These *rd10* and *Q344ter* models shared a similar pathogenesis with the *rd1* mice, in which the rod death resulted in secondary cone loss. Intriguingly, the cone loss in the *rd10* and *Q344ter* mutants was also alleviated by treatment with the same drug mixture. This treatment even preserved the cone function in *rd10* mice. These initial studies have therefore convincingly demonstrated the feasibility of prolonging visual function in retinal degeneration by drug treatment. Nonetheless, promising drug candidates are scarce.

The scarcity of new drugs has motivated research to discover novel compounds. One rich resource for novel drugs is traditional Chinese medicine (TCM) [6]. Many TCMs are theorized to benefit vision by restoring the balance of the body. In other words, they are prophylactic treatments. For example, *Lycium barbarum* polysaccharides (LBP) have been shown to protect retinal ganglion cells in a rat model of glaucoma [7], and to reduce retinal damage in a mouse model of retinal ischemia/reperfusion injury [8]. For many other TCMs, their possible eye benefits remain to be systematically characterized. One such example is the five-flavor fruit (*Fructus Schisandrae*). It has long been consumed by various populations for sustaining health and good vision [9]. These health benefits of the fruit are partially conferred by one of its active chemicals, Schisandrin B (SchB), as demonstrated by the following evidence. First, SchB protected cultured cardiomyocytes and rat hearts against oxidative injury [10–14]. Second, it inhibited P-glycoprotein and multidrug resistance-associated protein 1 [15,16], and in turn enhanced apoptosis of cancer cells under doxorubicin induction [17]. Third, it inhibited ataxia telangiectasia and Rad-3-related kinase (ATR), that controlled a cell-cycle check point [18]. The inhibition of ATR by SchB sensitized cancer cells to UV irradiation. These studies have demonstrated the prophylactic and therapeutic roles of SchB. Its eye benefits, however, have not been formally investigated.

We are inspired by the potential of TCMs and the aforementioned drug studies on RP mice. Hence, we evaluated in this study the prophylactic eye benefit of SchB in the zebrafish model. This vertebrate model has been widely used to study eye diseases [19–21] because a number of retinal-degeneration mutants are readily available. Among them, a *pde6c*^*w59*^ mutant is particularly suitable for testing SchB because of its similar genetic basis to a human retinal degeneration, and its unique pathogenesis. In the *pde6c*^*w59*^ mutant, an A>G point mutation was identified in the *pde6c* (*phosphodiesterase 6C*, *cGMP-specific*, *cone*, *alpha prime*) gene [22,23], two base pairs before exon 12 in the splice-acceptor site. This splice site mutation was predicted to create a frame shift in the coding sequence and result in a truncated protein or mRNA degradation through nonsense-mediated decay. A similar mutation was found in the human *PDE6C* gene in a positionally equivalent location (1483-2A>G) in patients suffering from achromatopsia [24], an eye condition whose phenotypes overlap those of cone dystrophy [24–27]. Consistent with the cone problems in patients, cones in the zebrafish *pde6c*^*w59*^ mutant began to degenerate at 4 days postfertilization (dpf) [22]. The cone death altered the local environment and created oxidative damage to the retina [28]. Consequently, the genetically-normal rods degenerated as bystanders [23,29]. Thus, the *pde6c*^*w59*^ PRs died due to either an intrinsic genetic mutation or as an indirect consequence of the changing environment, a pathogenesis similar to the aforementioned mouse RP models. This resemblance makes *pde6c*^*w59*^ a suitable model for testing the possible prophylactic effect of SchB, akin to the testing of the chemical mixtures in these mouse models [4,5].

In this study, SchB was tested on the *pde6c*^*w59*^ mutants. Their visual performance was determined by the visual motor response (VMR) [30,31], an assay that measured larval locomotion in response to an abrupt change in light illumination. The results show that prophylactic SchB treatment enhanced the VMR of *pde6c*^*w59*^ mutants towards light onset, and that the effect was substantially mediated through eye function. Furthermore, SchB specifically reduced the size of the abnormally large rods. Together, our study has successfully unveiled a possible visual benefit of SchB on a retinal-degeneration model.

## Materials and Methods

### Zebrafish maintenance and breeding

The *pde6c*^*w59/+*^ mutant line [22] was purchased from the Zebrafish International Resource Center (ZIRC) and maintained according to standard procedures [32,33]. When this line was raised, only the heterozygous carriers and their wild-type (WT) siblings would survive to adulthood. These heterozygous carriers were crossed to obtain homozygous mutant embryos (i.e. *pde6c*^*w59/w59*^), whereas their genotyped WT siblings were bred to generate WT embryos for control experiments. All collected embryos were raised in E3 medium [34] in a 28°C incubator with the same day-night cycle (14 h light / 10 h dark) as in the fish facility. The medium was changed every day. During the process, unhealthy embryos were discarded. All protocols were approved by the Purdue Animal Care and Use Committee.

### Genotyping

Zebrafish genotyping was conducted based on a previously reported protocol [23]. In short, a fragment of the *pde6c* gene was amplified by PCR with a specific pair of primers: pde6cF—TTGGCCTCTGGAATACTGGCTCTC; pde6cR—GTTTGACCAGAACCCGGAAG. The resulting PCR product was 157 base pairs (bp) long. During the amplification, a restriction site for BsaXI was created in the PCR product of the mutated *pde6c*^*w59*^ allele but not in that of the WT allele. These PCR products were then restricted by BsaXI and analyzed by agarose gel electrophoresis. On the gel, a WT allele would migrate as a single 157-bp band, whereas the *pde6c*^*w59*^ allele would be restricted by BsaXI, and would migrate as a 122-bp band and a 35-bp band.

### SchB preparation and animal treatment

SchB was extracted and purified from *Fructus Schisandrae* as previously reported [35]. The purified SchB powder was dissolved in DMSO and stored as a 7.5 mM stock solution at room temperature. During an experiment, the stock solution was diluted to the desired working concentration with E3 medium. For the treatment control, an equal volume of DMSO was diluted in the E3 medium. These solutions were used to treat zebrafish embryos from 3 to 6 dpf. During the course of treatment, the media was changed every day.

### Glucose-6-phosphate dehydrogenase (G6pd) assay

An enzymatic assay [36] was used to measure the specific activity of G6pd. This enzyme catalyzes the first rate-limiting step of the pentose phosphate pathway. In particular, it oxidizes glucose 6-phosphate (G6P) while reducing nicotinamide adenine dinucleotide phosphate (NADP+) to NADPH. In the assay, the rate of NAPDH formation was measured in a known amount of protein extract to determine the specific G6pd activity. The proteins were extracted from larvae by grinding them in a buffer containing 0.1 M Tris HCl pH 7.6, 10 mM MgCl_2_, and 1% 2-mercaptoethanol in a microcentrifuge tube. The resulting lysate was centrifuged to obtain the supernatant. To begin the G6PD assay, 100 μL of this supernatant was mixed with 1 mM G6P and 0.2 mM NADP+ to initiate NADPH production. Then, the NADPH produced in the reaction was determined by measuring the absorbance of the reaction mix at 340 nm every minute for 15 minutes in a Shimadzu UV1700 UV-Visible Spectrophotometer (Shimadzu, Kyoto, Japan). These measured absorbance values were compared with the absorbance of a known NADPH standard to calculate the amount of NADPH produced per min by the supernatant. Furthermore, the amount of protein in the supernatant was separately determined by the standard Bradford assay. Using these values, the specific G6pd activity was calculated by dividing the amount of NADPH produced per min with the measured protein amount.

### Optokinetic response (OKR) assay

In this study, a custom-made OKR apparatus was constructed based on the basic specifications reported in a previous study [37]. To conduct an OKR measurement, the larvae were partially immobilized in 3% methylcellulose in a 35-mm Petri dish. The dish was placed in the center of a circular drum with 20° black and white vertical stripes attached on the inner surface. These stripes were illuminated by a Fiber Lite M1-150 illuminator (Dolan-Jenner Industries, Boxborough, MA). The illuminance was approximately 20,000 Lux at the level of the Petri dish, as measured by a LX1010B light meter (Mastech, Taipei, Taiwan). During the OKR measurement, the rotation speed of the drum was set at eight revolutions per minute. In response to stripe rotation, normal larval eyes move in saccades [37]. This movement was independently evaluated by two individuals under a stereomicroscope to ensure accurate larval classification.

### Visualmotor response (VMR) assay

The VMR assay was based on a published design [30,31]. The assay was conducted inside a ZebraBox system (ViewPoint Life Sciences, Lyon, France), with larvae individually arranged in a Whatman 96-squarewell UNIPLATE (GE Healthcare Bio-Sciences, Marlborough, MA). Inside the system, animals were isolated from environmental light and stimulated by white light emitted by a light-controlling unit from the bottom of the plate. The larval movement was recorded by an infra-red camera at a rate of 30 frames per second under 850-nm infrared illumination, which the animals could not perceive. Before the actual experiment, the 96-well plate with the larvae was dark-adapted in the ZebraBox system for 3.5 hours to acclimatize the animals. The actual test consisted of three consecutive trials of light onset (Light-On) and light offset (Light-Off) periods, with each period lasting for 30 minutes (S1 Fig). The light change (On or Off) was abrupt and instantaneous. The Light-On stimulus was set at 100% of the output intensity of the ZebraBox. The total irradiance from 300 nm to 750 nm was measured by an EPP2000 Spectrometer (StellarNet Inc, Tampa, FL) at nine evenly distributed locations across the surface of the light-controlling unit that would be covered by the 96-well plate. The mean ($\bar{x}$) total irradiance was 1.24 W m^-2^, and the standard deviation (*s*) of the measurement was 0.20 W m^-2^. Larval activity during the VMR assay was recorded by Zebralab software (ViewPoint Life Sciences, Lyon, France) running in the quantification mode. The following parameters were used to collect activity data: detection sensitivity per pixel per image—grey level 6; burst threshold—4 pixels; bin size—1 second. The detection sensitivity registered pixels with a grey level below a preset level. These registered pixels detected the individual larvae in each frame. If these pixels were detected in a different location in successive frames, they were declared as active pixels. These active pixels represented the part of the larvae that moved in successive frames. The burst threshold selected movements that were larger than a predefined number of active pixels between successive frames to separate small movements from major movements. Larval movement was summarized as the fraction of frames that a larva displayed movement in each second (as defined by the bin size). This fraction was defined as the Burst Duration, and was individually computed for all larvae. VMR assays were started at around the same time at 2 p.m. in the day to minimize the effect of circadian rhythm on vision [38]. The media were changed every day. The larval genotype was confirmed by PCR after all behavioural experiments.

### Enucleation

Enucleation was performed on 5-dpf larvae. First, they were anaesthetized with 0.03% Tricaine (Sigma-Aldrich, St. Louis, MO) in Ringer’s solution [39] for immobilization. Then, their eyes were removed by a fine tungsten-wire loop etched as described [39]. After enucleation, the larvae were kept in Ringer’s solution at 28°C for one hour to recover. Finally, they were transferred back to the original treatment medium overnight and subjected to the VMR assay at 6 dpf.

### Immunohistochemistry of dissected retinas

To conduct retinal immunostaining, retinas were microdissected from 6-dpf larvae fixed in 4% paraformaldehyde (PFA) at room temperature for two hours. The dissection removed the adhering sclera and retinal pigment epithelium (RPE), which was critical for antibody penetration. To dissect retinas, a larva was held dorsal side up on a Petri dish using Dumont #55 forceps. Then, the RPE-attached retina was detached from the sclera by dissecting through the lateral side of the eye with a fine hook created by bending a tungsten needle, etched as described previously [39, 40](see S2B Fig). For the lateral-side dissection, the hook was inserted into the anterior chamber by puncturing the cornea. The tip of the hook would slide beneath the scleral region just outside the pupil (S2A Fig, black circular arrow). Then, the RPE-attached retina could be easily detached from the sclera by a gentle push from the medial side of the eye (S2A Fig, white arrow). Retinas were washed in a microcentrifuge tube once with 0.5 mL 1X phosphate-buffered saline/0.1% Tween 20 (PBST) for 10 mins, and twice with Milli-Q water for 5 mins each. They were then incubated in 0.5 mL acetone for 20 mins at -20°C to aid penetration and to detach the RPE. After RPE detachment, the tissues were washed twice with 0.5 mL Milli-Q water for 5 mins each, and thrice with 0.5 mL 1X PBST for 15 mins each to remove residual acetone. The detached RPE could be easily removed from the retina by a tungsten needle or forceps (S2B Fig). The dissected retinas were kept in a microcentrifuge tube for immunostaining (S2C Fig).

For immunostaining, tissues were blocked (10% goat serum, 1% bovine serum albumin [BSA], and 1% Triton-X 100 in 1X PBST) at room temperature for 2 hours and incubated at 4°C overnight in incubation buffer (1% goat serum, 1% BSA, and 1% Triton-X 100 in 1X PBST) with primary antibodies: mouse anti-zpr1 (1:200) for red/green double cones [41] or anti-4c12 for rods [22]. After rinsing three times for 30 mins each in washing buffer (1% Triton-X 100 in 1X PBST), tissues were incubated with secondary antibody, Alexa Fluor 488 goat anti-mouse IgG (1:1000), in incubation buffer at 4°C overnight, then rinsed three times with 1X PBST for 30 mins each. During all incubation steps, the microcentrifuge tubes were gently shaken on a rotary platform. Stained retinas were mounted on a glass slide in 70% glycerol/1X PBST, covered with a cover slip elevated by small droplets of vacuum grease, and imaged with a Zeiss LSM-710 Confocal Microscope (Carl Zeiss, Thornwood, NY) using the z-stack module.

### Confocal image analysis

The z-stack confocal images were trimmed in ZEN black edition version 8.1 (Carl Zeiss, Thornwood, NY) to include the central-retina region immediately dorsal to the optic nerve. This region contained the more mature PRs, which were substantially degenerated in the *pde6c*^*w59*^ mutant [22]. As a result, analyzing these central-retina PRs would maximize the chance of detecting SchB’s effect on degenerating *pde6c*^*w59*^ PRs. For zpr1-stained and 4c12-stained retinas, the analyzed areas were 19413 and 9089.9 μm^2^, respectively. The PR number in these regions was manually counted in Vaa3d [42,43], whereas PR morphology was analyzed using 3D ImageJ [44] implemented in Fiji <http://fiji.sc/Fiji>.

The following procedure was used to conduct 3D analysis. First, PRs were segmented as regions of interest (ROIs) using the 3D object counter with the default thresholds. These segmented ROIs were then inspected in RoiManager 3D to select only those ROIs that covered a complete PR, or those that could be split or merged into a complete PR. ROIs were excluded if their morphology was distorted by proximity to the optic nerve region. Finally, the selected ROIs were used to measure several morphological parameters, including volume, sphericity, Feret diameter, ratios between the radii of a fitted 3D ellipsoid, and ratio of the volume of the fitted 3D ellipsoid to the measured volume.

### Statistical analysis

Statistical analyses were performed in R version 3.2.0 <http://www.r-project.org>. G6pd activity was analyzed by Welch two-sample *t-*test. OKR counts were analyzed by Pearson’s Chi-squared test. VMR data were processed by DataWorkShop (ViewPoint Life Sciences) to extract locomotor activity from 60 s before to 60 s after each light-stimulus change. Extracted data were plotted to show average activity and corresponding standard error under the same light stimulus in every second. To compare activity profiles, the locomotor activity from different conditions was segregated into two time periods: before light change (-29–0 s) and after light change (1–30 s). The period 31–60 s after light change was sometimes also analyzed. The activity profiles in these time periods were compared by the Hotelling’s *T*^*2*^ test [45–47] implemented in the *Hotelling* package in R. For each comparison, five thousand permutations were performed to estimate the null distribution for *p-*value calculation. Finally, the resulting *p*-values were corrected for multiple-hypothesis testing by controlling the false discovery rate. For immunostaining results, the PR count data were analyzed by Welch two-sample *t-*test for two-group comparison or Kruskal-Wallis rank sum test for three-group comparison. The PR morphological data were analyzed by linear mixed-effects modeling, with individual retinas modeled as a random effect.

## Results

### Optimizing a SchB concentration for treating zebrafish larvae

**Fig 1:**
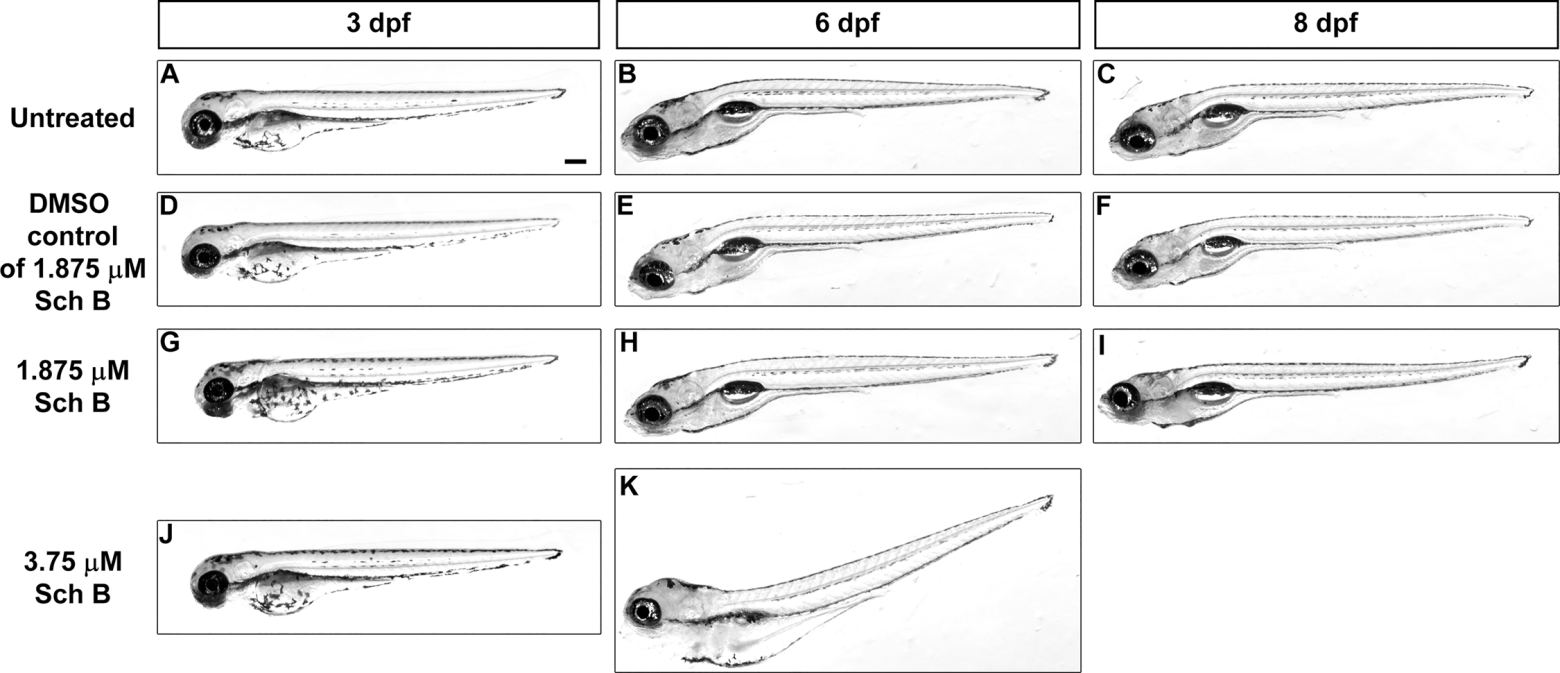
Optimization of SchB concentration for treating zebrafish larvae. The gross morphology of zebrafish larvae under different treatment schemes from 3 to 8 dpf. (A–C) untreated controls; (D–F) larvae exposed to the same volume of DMSO carrier as in the 1.875 μM SchB treatment; (G–I) larvae exposed to 1.875 μM SchB; (J–K) larvae exposed to 3.75 μM SchB. In each treatment group, the lateral view of a representative larva at 3, 6 and 8 dpf is shown. Larvae exposed to higher SchB concentrations, including 7.5 and 15 μM, died relatively rapidly (Table 1); hence, these samples are not shown here. Scale bar = 200 μm.

To study SchB effect in the zebrafish system, a treatment concentration had to be determined. The effective concentrations of SchB have been defined in several other biological systems before [10–18]. For example, a known antioxidant response was induced in cultured rat cardiomyocytes by exposure to 15 μM SchB for six hours, or to 2.5–7.5 μM SchB for 24 hours [10,14]. These conditions served as guidelines for optimizing a SchB concentration for treating zebrafish larvae. A dilution series of SchB (15, 7.5, 3.75 and 1.875 μM) was prepared in E3 medium. Each dilution was used to treat 60 WT larvae (Fig 1A–1K, Table 1). In the control group, 60 WT larvae were exposed to E3 medium supplemented with the same amount of DMSO carrier as in the 1.875 or 15 μM SchB dilutions (i.e. 0.025% or 0.2% DMSO). The larvae were treated starting at 3 dpf, a stage when the first visual response appeared [48,49], and the normal Pde6c protein would be functional to transduce light signal. Starting the treatment at this stage would minimize any unwanted drug effects on early retinogenesis of the treated larvae. Larvae were then observed daily until 8 dpf.

**Table 1:** The survival counts of zebrafish larvae after exposure to various concentrations of SchB from 3 to 8 dpf. Normal WT embryos were exposed to 1.875, 3.75, 7.5 and 15 μM of SchB or DMSO carrier as controls. The treatment took place from 3 to 8 dpf. The survival of larvae in each condition was counted every day. The DMSO percentage in the controls was 0.025% and 0.2%. These were the corresponding DMSO amounts used as the drug carrier in treatment groups with 1.875 and 15 μM SchB respectively. Both DMSO percentages were lower than the commonly used percentage (< 0.3%) in other zebrafish drug studies [60,61]. Hence, the use of these specific amounts of DMSO should not affect the survival of the larvae. Indeed, they did not. In each experimental group, 60 embryos were used. Then, the survival was counted every day from 3 to 8 dpf. The 3-dpf count was obtained immediately after the addition of chemicals.

Sample	Count					
	3 dpf	4 dpf	5 dpf	6 dpf	7 dpf	8 dpf
Untreated	60	60	60	59	59	59
0.025% DMSO (*Control of 1*.*875 μM SchB*)	60	60	60	60	60	60
0.2% DMSO (*Control of 15 μM SchB*)	60	60	60	60	60	60
1.875 μM SchB	60	60	60	60	60	58
3.75 μM SchB	60	60	60	60	0	0
7.5 μM SchB	60	60	60	12	0	0
15 μM SchB	60	0	0	0	0	0

Larvae exposed to 15 μM SchB died quickly by 4 dpf, whereas those exposed to 7.5 and 3.75 μM SchB survived well until 5 and 6 dpf, respectively (Table 1), and then died usually by the next day. The 3.75 μM-SchB group suffered from obvious morphological defects at 6 dpf before they died (Fig 1K). On the contrary, those treated with 1.875 μM SchB survived well until 7 dpf (Table 1), and the majority that survived until 8 dpf (96.6% [58/60]) had normal morphology (Fig 1I). Throughout treatment, DMSO-treated controls looked identical to untreated larvae (Fig 1A–1F) and had a comparable survival rate at 8 dpf (DMSO-treated controls: 100% [60/60] for both 0.025% and 0.2% DMSO; untreated controls: 98.3% [59/60]; Table 1). Thus, 1.875 μM SchB neither disturbed morphology nor increased mortality.

To determine whether 1.875 μM SchB was pharmacologically active in these larvae, an enzymatic assay of G6pd [36] was performed since SchB was known to activate Glucose-6-phosphate dehydrogenase (G6pd) [14]. Ten WT larvae were exposed to either 1.875 μM SchB or an equal volume of DMSO from 3 to 6 dpf. This treatment time was chosen for our ultimate characterization of prophylactic property of SchB on the *pde6c*^*w59*^ PRs, which began to degenerate at 4 dpf [22]. Three independent biological replicates were collected, and G6pd activity was measured by NADPH production in the presence of G6P and NADP+. G6pd specific activity was substantially enhanced in the SchB-treated group compared with that in the DMSO-treated controls (0.21 ± 0.03 vs. 0.074 ± 0.02 μM mg^-1^ min^-1^, Welch two-sample *t-*test *p* = 0.0023). These results suggest that the known pharmacological effect of SchB could be induced by exposing the embryos to 1.875 μM SchB from 3 to 6 dpf (designated as the SchB treatment hereafter).

### The SchB treatment significantly enhanced the visual motor response (VMR) that was attenuated in the *pde6c*^*w59*^ mutants

The SchB treatment was then tested on the *pde6c*^*w59*^ mutants. In particular, their potential change in visual performance was evaluated by VMR, a primitive startle response displayed by the larvae within seconds after light onset and offset [30]. This response was visually-evoked, as it is absent in an eyeless mutant *chokh/rx3* [30,31]. Hence, this VMR assay was used to evaluate the light sensation of the *pde6c*^*w59*^ mutants under different conditions.

**Fig 2:**
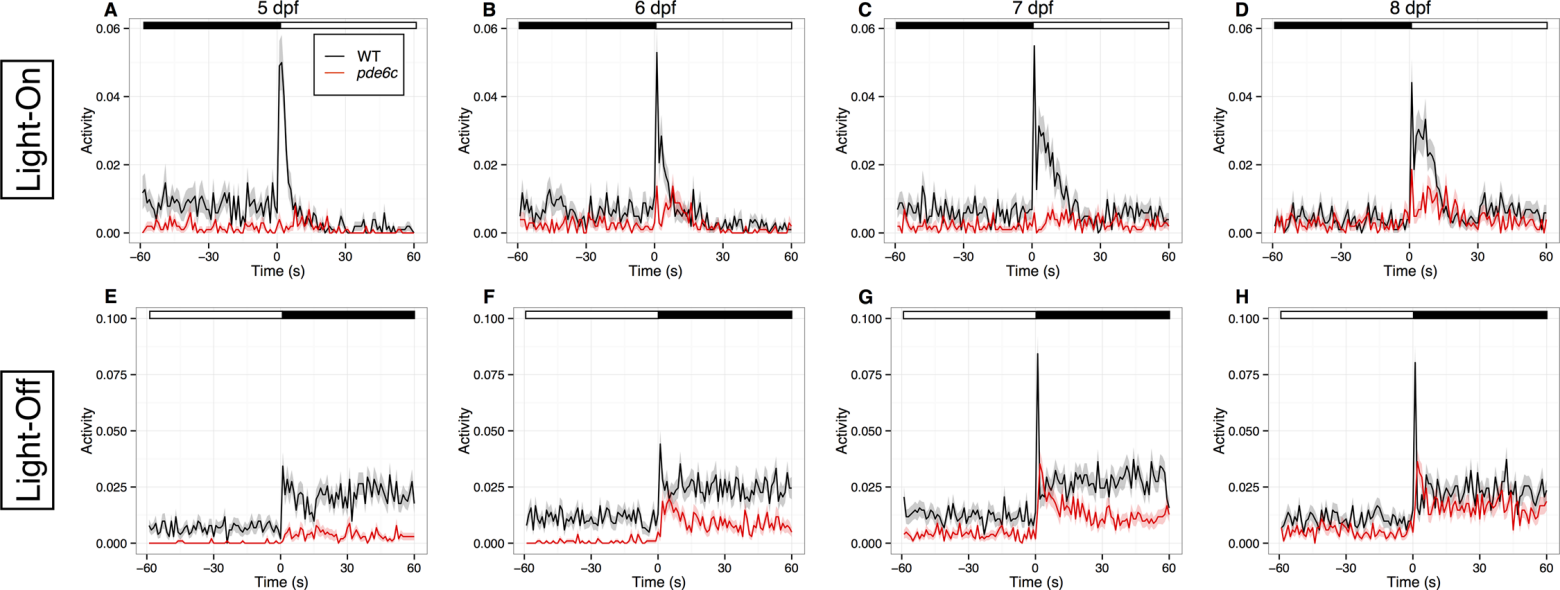
The *pde6c*^*w59*^ larvae displayed an attenuated VMR. (A–H) The activity plots of WT larvae (black traces; N = 34) and *pde6c* ^*w59*^ larvae (red traces; N = 34) from 5 to 8 dpf. Figures A–D show the Light-On VMR, whereas Figures E–H show the Light-Off VMR. The VMR assay consists of three consecutive trials of a Light-On and a Light-Off stimulus (S1 Fig; also see methods). Each stimulus would last for 30 mins. During the experiment, the larval movement was recorded by computer as movement duration per second. Then, the activity of the same type of larvae was averaged across the three Light-On or Light-Off trials and plotted in the figures. The solid traces in each plot show the mean activities from 60 s before light change to 60 s after light change, whereas the ribbons surrounding these activity traces indicate the corresponding standard error of the mean. At the top of the plots, the white and black bars indicate light and dark phases respectively. These plots show that the *pde6c*^*w59*^ larvae displayed a substantially attenuated VMR compared with the WT larvae. The problem was likely caused by the retinal degeneration in the *pde6c*^*w59*^ mutants, which affected their capability to sense light change in the environment. The raw data for Fig 2 are available in S1 File.

First, the VMR assay was used to evaluate the difference between the untreated *pde6c*^*w59*^ mutants and the WT controls. In this experiment, the mutant larvae were identified by their lack of optokinetic response (OKR) at 5 dpf, a response that became robust at this stage [37,49]. This is also the earliest stage to identify a large number of mutants by a non-invasive approach. Specifically, 34 OKR-negative larvae and 34 WT controls were individually arranged in the wells of a 96-well plate. Then, their VMR was measured from 5 to 8 dpf (Fig 2). The VMR was significantly different between these larvae. The WT larvae displayed a drastically increased VMR within a few seconds after light onset (Light-On) at all stages (Fig 2A–2D, black traces). From 6 to 8 dpf, smaller activity peaks were also prominent after the main peak. At all stages, the activity rapidly returned to baseline in approximately 15–20 s. When subjected to the light offset (Light-Off), the WT activity was substantially increased in the first 5–10 s (Fig 2E–2H, black traces). By 15 s, it was reduced to an intermediate level and sustained for an extended period of time. This sustained activity would gradually return to baseline before the end of the Light-Off period at 30 mins (i.e. the dark periods in Fig 2A–2D). Compared with the WT controls, the *pde6c*^*w59*^ larvae displayed a significantly attenuated VMR at all stages (Fig 2; red traces). Their Light-On VMR was much smaller at 6 and 8 dpf, and was barely noticeable at 5 and 7 dpf (Fig 2A–2D); whereas their Light-Off VMR became gradually larger from 5 to 8 dpf (Fig 2E–2H). The *pde6c*^*w59*^ activity profiles differed significantly from those of WT larvae from 1 to 30 s after light change (Hotelling’s *T*^*2*^ test; *p* < 0.0001) at all stages under both Light-On and Light-Off stimuli.

The VMR assay was used to evaluate the effect of SchB treatment on light sensation. Mutant larvae were exposed to the SchB treatment (i.e. 1.875 μM) or corresponding amount of DMSO (0.025%) starting at 3 dpf. At this stage, the mutants could not be reliably identified by any non-invasive approach. They were rather identified at 5 dpf by their lack of OKR as discussed before. The identification was possible because the SchB treatment did not induce an OKR in the *pde6c*^*w59*^ mutants, as demonstrated by the following independent experiment: The embryos obtained from a heterozygous cross were randomly divided into two groups. At 3 dpf, one group of embryos (N = 522) was exposed to 1.875 μM SchB, while the other group of embryos (N = 582) was exposed to the same amount of DMSO carrier (0.025%). Their OKR was then measured at 5 dpf. If a response could be induced by the SchB treatment in the mutants, the fraction of OKR-negative larvae would be significantly reduced in the SchB-treated group but not in the DMSO-control group. The results suggest this was not the case. In the SchB-treated group, 116 out of 522 larvae (22.2%) did not display an OKR; whereas in the DMSO-control group, 132 out of 582 larvae (22.7%) did not display a response. The fraction of the OKR-negative larvae was not different between these two groups (Pearson Chi-squared test; *p* = 0.91). The *pde6c* genotype of these larvae was also confirmed by PCR of randomly sampled individuals from the experimental groups. The true positive rate (i.e. genotyped homozygous mutant) was comparable between the SchB-treated group (13/15 or 86.7%) and the DMSO-control group (15/15 or 100%) (Fisher’s exact test; *p* = 0.48). Thus, SchB treatment did not induce an OKR in the *pde6c*^*w59*^ larvae, and the OKR assay could be used to identify these mutants with or without SchB treatment.

**Fig 3:**
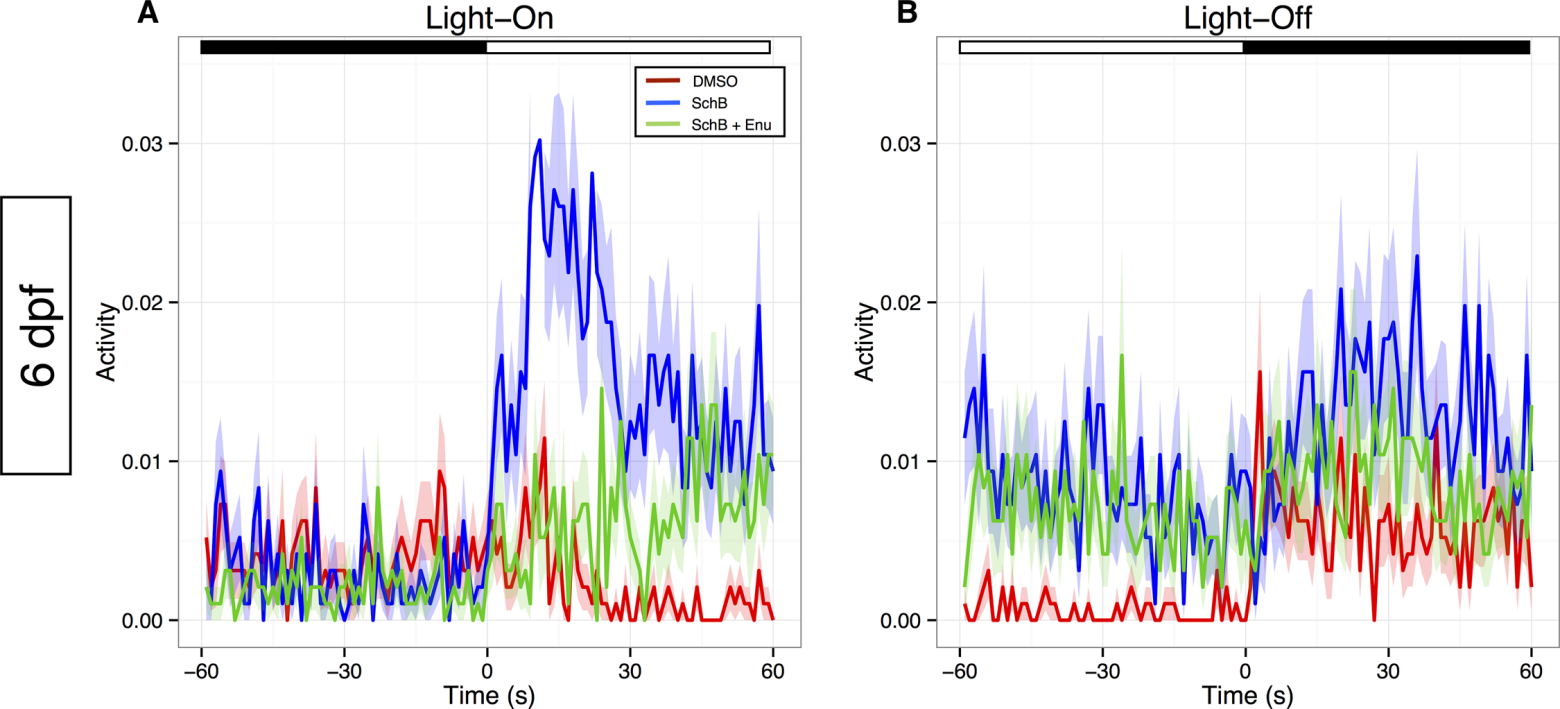
The SchB treatment enhanced the VMR of *pde6c*^*w59*^ larvae through their eyes. (A & B) The activity plots of 6-dpf *pde6c*^*w59*^ larvae that were exposed to three different treatments: (1) a three-day treatment of 1.875 μM of SchB (blue traces; N = 32); (2) a three-day treatment of 0.025% DMSO carrier, the same amount as in the SchB treatment (red traces; N = 32); and (3) the same SchB treatment as in condition (1), except for the enucleation of the larval eyes at 5 dpf (green traces; N = 32). Figure A shows the Light-On VMR of these larvae, whereas Figure B shows their Light-Off VMR. The VMR assay was run as described in Fig 2 and S1 Fig. In each plot, the solid traces show the mean activities from 60 s before light change to 60 s after light change, whereas the ribbons surrounding these activity traces indicate the corresponding standard error of the mean. The same plots without the error ribbons are shown in S3 Fig to emphasize just the average traces. At the top of these plots, the white and black bars indicate light and dark phases. The results reveal that the SchB treatment enhanced the Light-On VMR of the *pde6c*^*w59*^ larvae (blue traces vs. red traces) and that this enhancement was substantially attenuated by eye enucleation (A; green trace). Together, these observations suggest that SchB enhanced the response of *pde6c*^*w59*^ larvae to light onset through their eyes. The raw data for Fig 3 are available in S2 File.

In the VMR experiment, these OKR-negative larvae were identified at 5 dpf and individually arranged in the wells of a 96-well plate that contained the corresponding treatment medium. After acclimatizing overnight, their VMR was analyzed at 6 dpf (Fig 3). In the SchB-treated group, the Light-On VMR was substantially enhanced compared with that of the DMSO-treated group (Fig 3A; blue trace vs. red trace, N = 32 in each group; Hotelling’s *T*^*2*^ test, 1 to 30 s after light onset; *p* < 0.0001). This VMR enhancement induced by SchB was not a general excitation of the larvae but rather an effect specifically associated with light onset. This is supported by the comparison of VMR data between the time periods before light onset (-29–0 s) and after light onset (1–30 s) (Hotelling’s *T*^*2*^ test; *p* < 0.0001). For the Light-Off VMR, the SchB-treated group displayed a higher activity during the sustained phase of the response compared with the DMSO-treated group (Fig 3B; blue trace vs. red trace), while the acute peak response was diminished. Nonetheless, the comparison of the VMR data from 1 to 30 s was not significant (Hotelling’s *T*^*2*^ test; *p* = 0.10). At the end of the VMR experiments, all larvae were also genotyped to confirm their identity. Hence, these results indicate that the attenuated Light-On VMR of *pde6c*^*w59*^ mutants was enhanced by the SchB treatment.

### SchB enhanced the VMR of *pde6c*^*w59*^ mutants through their eyes

The SchB enhancement of *pde6c*^*w59*^ mutants’ VMR suggests that the mutants sensed light change better, possibly through their eyes. To test this hypothesis, a separate group of SchB-treated *pde6c*^*w59*^ larvae were enucleated before the VMR assay (Fig 3A & 3B, green traces; N = 32). The analysis indicates that the Light-On VMR of the enucleated *pde6c*^*w59*^ larvae with SchB treatment was significantly attenuated compared with those with intact eyes (Fig 3A, green vs. blue traces; Hotelling’s *T*^*2*^ test, 1–30 s after light onset; *p* < 0.0001). It was also noted that the activity of the enucleated SchB-treated *pde6c*^*w59*^ larvae gradually increased and reached a similar level as those with intact eyes from approximately 40 s onwards. Indeed, there is no difference in the VMR between these two groups from 31 to 60 s (Hotelling’s *T*^*2*^ test; *p* = 0.27). This activity increase was not observed in the DMSO-treated controls (Fig 3A, red trace). During light offset, the enucleated *pde6c*^*w59*^ larvae with SchB treatment displayed a highly similar Light-Off VMR to those with intact eyes (Fig 3B, green vs. blue traces; Hotelling’s *T*^*2*^ test, 1 to 30 s after light offset; *p* = 0.53). Together, the eye-enucleation test suggests that SchB enhanced the first 30 s of Light-On VMR of the *pde6c*^*w59*^ larvae through their eyes.

### The SchB treatment reduced the size of the abnormally-large *pde6c*^*w59*^ rods

**Fig 4:**
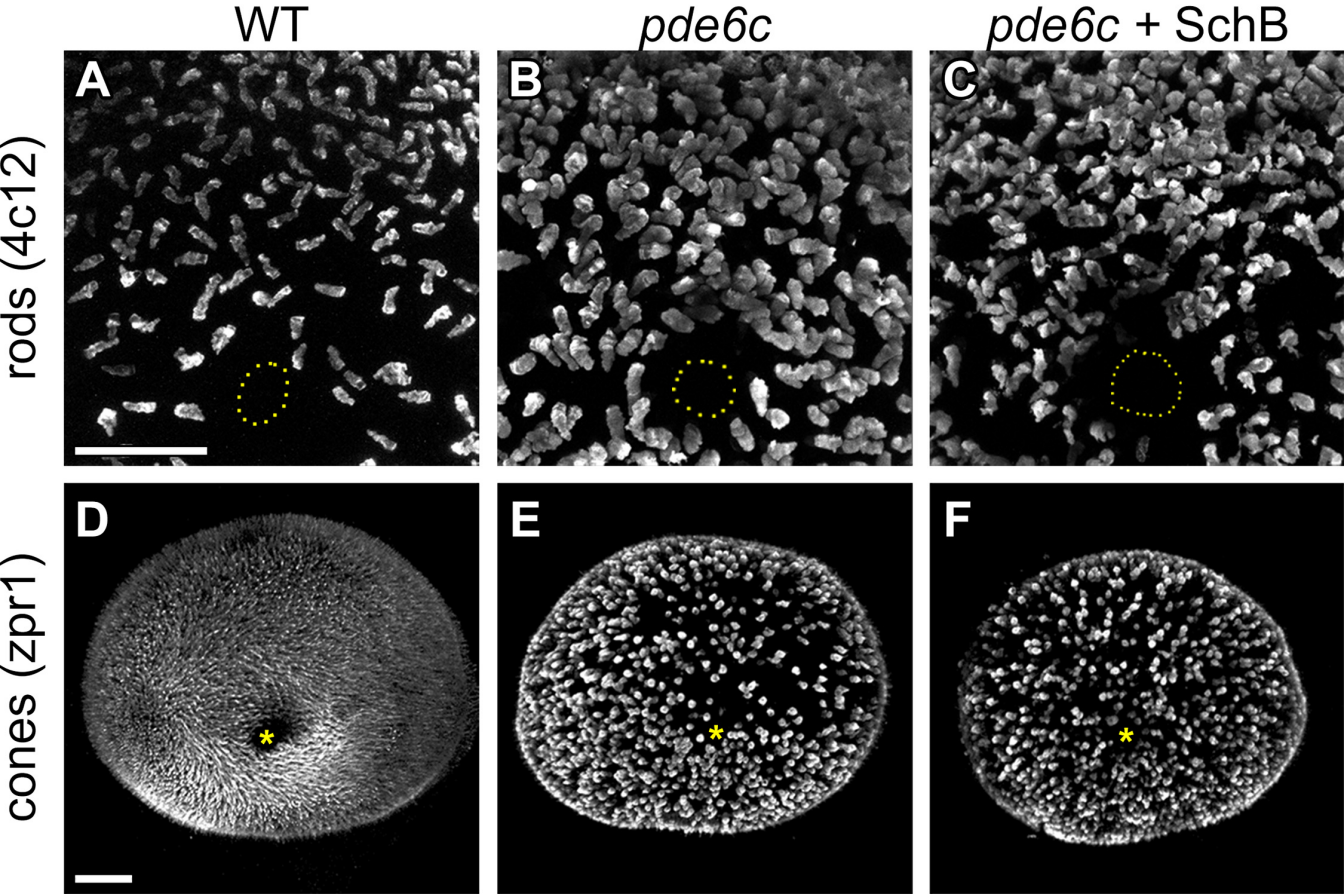
Photoreceptor morphology in the *pde6c* ^*w59*^ retinas with and without SchB treatment. Whole-mount immunostaining was conducted to evaluate the effect of SchB treatment on rods (A–C) and cones (D–F) in the *pde6c*^*w59*^ mutants. The rods were immunostained with anti-4c12 [22], whereas the red-green double cones were immunostained with anti-zpr1 [41]. In (A–C), a central-medial region of the retina is shown, and the optic nerve is highlighted by a dotted circle. In (D–F), the medial view of the whole retina is shown, and the optic nerve is indicated by a yellow asterisk. In all figures, dorsal is to the top. Scale bars = 50 μm.

Since SchB enhanced the attenuated Light-On VMR of the *pde6c*^*w59*^ mutants through their eyes (Fig 3), the drug might act on their degenerating PRs. To evaluate this possibility, PR immunostaining was conducted on retinas dissected from 6-dpf *pde6c*^*w59*^ larvae that were exposed to SchB or DMSO (Fig 4), using the same treatment scheme as in the VMR experiment above. In addition to the *pde6c*^*w59*^ larvae, stage-matched WT larvae were treated with DMSO and used as an internal control. All these samples were analyzed by anti-4c12 for rods [22] and anti-zpr1 for red-green double cones [41,50,51]. The results show that the SchB treatment reduced the size of the abnormally-large rods; however, it did not have an effect on the *pde6c*^*w59*^ cones.

Without the SchB treatment, the *pde6c*^*w59*^ rods generally appeared more prominent (Fig 4B) than the WT ones (Fig 4A), a phenomenon that had been previously described [22]. To determine the specific morphological changes between them, several morphological parameters and cell count were measured. Two of these parameters were statistically different: cell volume and the ratio between the second & third radii of the fitted 3D ellipsoid (*p* = 0.0013 and 0.0019 respectively; Table 2). The volume of the *pde6c*^*w59*^ rods was larger than that of the WT rods, which confirms Stearns and colleagues’ observation. This volume increase was partially explained by a decrease in the ratio between the second & third radii of the fitted 3D ellipsoid, a change that indicates the *pde6c*^*w59*^ rods were rounder than the WT rods. The remaining parameters, and cell count, were not statistically different between the *pde6c*^*w59*^ and WT rods. Under the SchB treatment, the *pde6c*^*w59*^ rods became significantly smaller (Fig 4C) compared with the untreated ones (Fig 4B) (*p* = 0.0008; Table 2). The other morphological parameters and cell counts were not significantly different between the *pde6c*^*w59*^ rods with and without SchB treatment. For cones, they were obviously more prominent and less in number in the *pde6c*^*w59*^ mutants without the SchB treatment (Fig 4E) compared with those in the WT controls (Fig 4D). Unlike rods, cone morphology or count in the *pde6c*^*w59*^ mutants was not substantially altered by the SchB treatment (Table 3). Together, these results suggest that SchB treatment likely acted on the degenerating *pde6c*^*w59*^ rods and reduced their abnormally-large volume to the WT level.

**Table 2:** Rod count and morphological parameters measured from *pde6c* ^*w59*^ retinas with and without SchB treatment. *Pde6c* ^*w59*^ retinas were microdissected from 6-dpf larvae. These larvae were either exposed to DMSO carrier (*pde6c* DMSO) or SchB (*pde6c* SchB), under the same conditions that induced a positive effect on the Light-On VMR. WT retinas were included as an internal control to compare with the *pde6c* DMSO, as a previous study indicated a qualitative volume increase in the *pde6c* ^*w59*^ rods [22]. All these retinas were subjected to immunostaining with the rod marker 4c12 [22] and imaged by confocal microscopy (Fig 4A–4C). Then, cell count and morphological analyses of rods were conducted in a central-retinal region on the dorsal side of the optic nerve. This mature region of the retina displayed the most prominent rod degeneration in Stearns and colleagues’ study. Analyzing this region would maximize the chance to detect morphological changes. For cell-count analysis, 4, 6, and 8 retinas were used for WT, *pde6c* DMSO and *pde6c* SchB respectively. From these retinas, rods were segmented from the 3D confocal z-stacks using parameters outlined in the methods. The subsequent morphological analyses used only those individual rods that could be successfully segmented from the images and were not distorted by their proximity to the optic nerve. The total rod numbers used in the analyses were 116, 30, and 101 for WT, *pde6c* DMSO and *pde6c* SchB respectively. The *p-*values less than 0.05 are highlighted in boldface. The results not only confirm that *pde6c* ^*w59*^ rods were larger than WT rods as previously observed by Stearns and colleagues, but also reveal that the SchB treatment reduced the abnormal volume of *pde6c* ^*w59*^ rods to a level similar to WT. Furthermore, SchB did not affect other morphological parameters and cell count of the *pde6c* ^*w59*^ rods. The raw data for Table 2 are available in S3 File.

	Sample		Statistical comparison	Sample	Statistical comparison
**Parameter(unit)**	**WT(mean ± SD)**	***pde6c* DMSO(mean ± SD)**	***pde6c* DMSO vs. WT*p-value***^1^	***pde6c* SchB(mean ± SD)**	***pde6c* DMSO vs. *pde6c* SchB *p-value***^1^
Count (per 1000 μm^2^)	7.48 ± 0.32	9.39 ± 2.15	0.26	10.96 ± 2.30	0.31
**Morphological parameter [variable name in Fiji] (unit; if applicable)**	**WT (mean ± SE)**^**2**^	***pde6c* DMSO(mean ± SE)**^**2**^	***pde6c* DMSO vs. WT*p-value***^3^	***pde6c* SchB (mean ± SE)**^2^	***pde6c* DMSO vs. *pde6c* SchB *p-value***^3^
Volume [Vol_unit] (μm^3^)	135.63 ± 18.85	243.24 ± 19.72	**0.0013**	141.54 ± 14.41	**0.0008**
Sphericity [Spher_unit]	0.203 ± 0.029	0.262 ± 0.026	0.1425	0.314 ± 0.021	0.1337
Feret diameter [Feret_unit] (μm)	12.66 ± 0.59	13.19 ± 0.61	0.5452	11.93 ± 0.45	0.1180
Ratio between the major and second radii of the fitted 3D ellipsoid [Ell_Elon]	1.58 ± 0.06	1.54 ± 0.08	0.7040	1.75 ± 0.05	0.0517
Ratio between the second and third radii of the fitted 3D ellipsoid [Ell_Flatness]	1.71 ± 0.05	1.43 ± 0.06	**0.0019**	1.48 ± 0.04	0.4443
Ratio between the volume of fitted 3D ellipsoid and the volume of object[RatioVolEllipsoid]	0.51 ± 0.17	0.45 ± 0.14	0.7773	0.49 ± 0.12	0.7928

^1^The count data were analyzed by Kruskal-Wallis rank sum test. The *post hoc* pairwise multiple comparisons were done by Dunn’s test with Bonferroni adjustment.

^2^Mean and standard error (SE) were calculated from the corresponding coefficients of the fitted linear mixed-effects models.

^3^Contrast extracted from the fitted linear mixed-effects models.

**Table 3:** Cone count and morphological parameters measured from *pde6c* ^*w59*^ retinas with and without SchB treatment. *Pde6c* ^*w59*^ retinas were microdissected from the 6-dpf larvae. These larvae were either exposed to DMSO carrier (*pde6c* DMSO) or SchB (*pde6c* SchB), under the same conditions that induced a positive effect of the Light-On VMR. All these retinas were subjected to immunostaining with a cone marker zpr1 [41] and imaged by confocal microscopy (Fig 4D and 4E). Then, cell count and morphological analyses of cones were conducted in a central-retinal region on the dorsal side of the optic nerve in the *pde6c* ^*w59*^ retinas. WT cones were excluded from this statistical analysis because they were obviously different when compared with the *pde6c* ^*w59*^ cones. For cell-count analysis, 7 and 4 retinas were used for *pde6c* DMSO and *pde6c* SchB respectively. From these retinas, cones were segmented from the 3D confocal z-stacks using parameters outlined in the methods. The subsequent morphological analyses only used those individual cones that could be successfully segmented from the images and were not distorted by their proximity to the optic nerve. The total cone numbers used in the analyses were 247 and 121 for *pde6c* DMSO and *pde6c* SchB respectively. The results show that SchB treatment did not significantly change the count or morphology of the zpr1-positive cones in the *pde6c* ^*w59*^ mutants. The raw data for Table 3 are available in S4 File.

	Sample		Statistical comparison
**Parameter (unit)**	***pde6c* DMSO (mean ± SD)**	***pde6c* SchB (mean ± SD)**	***pde6c* DMSO vs. *pde6c* SchB *p-value***^1^
Count (per 1000 μm^2^)	7.68 ± 1.18	4.64 ± 2.35	0.075
**Morphological parameter [variable name in Fiji] (unit; if applicable)**	***pde6c* DMSO (mean ± SE)**^2^	***pde6c* SchB (mean ± SE)**^2^	***pde6c* DMSO vs. *pde6c* SchB *p-value***^3^
Volume [Vol_unit] (μm^3^)	405.49 ± 19.81	405.43 ± 26.92	0.9988
Sphericity [Spher_unit]	0.518 ± 0.008	0.524 ± 0.010	0.6379
Feret diameter [Feret_unit](μm)	16.26 ± 0.34	16.55 ± 0.45	0.6268
Ratio between the major and second radii of the fitted 3D ellipsoid [Ell_Elon]	1.987 ± 0.043	2.015 ± 0.059	0.7134
Ratio between the second and third radii of the fitted 3D ellipsoid [Ell_Flatness]	1.33 ± 0.023	1.30 ± 0.031	0.5157
Ratio between the volume of fitted 3D ellipsoid and the volume of object [RatioVolEllipsoid]	0.59 ± 0.11	0.63 ± 0.15	0.816

^1^Welch two-sample *t-*test

^2^Mean and standard error (SE) were calculated from the corresponding coefficients of the fitted linear mixed-effects models

^3^Contrast extracted from the fitted linear mixed-effects models

## Discussion

Retinal degeneration irreversibly impairs vision. Nonetheless, the underlying cell death is progressive [3]. This feature has provided a window through which prophylactic drug treatment may slow down the dying process and preserve visual function. This drug-intervention approach has been demonstrated by treating retinal degeneration models *rd1* and *rd10* with a chemical mixture [4,5]. Despite these encouraging findings, few effective compounds have been tested in this manner. In this study, we evaluated SchB on retinal degeneration induced by *pde6c* mutation in zebrafish. The results show that SchB enhanced the *pde6c*^*w59*^ mutant’s response to light onset in a VMR assay through a positive effect on the eye level (Fig 3). Furthermore, the SchB treatment also significantly reduced the volume of the abnormally-large *pde6c*^*w59*^ rods to the normal level (Fig 4 and Table 2). Thus, SchB likely exerted a positive effect on the degenerating *pde6c*^*w59*^ rods.

The *pde6c*^*w59*^ rods ultimately die as bystanders of cone death [22] through apoptosis [28]. This cell-death process can be triggered by several external factors, including loss of neighboring cell support [52], release of toxic factors from dying cells [53], light damage [54], and oxidative stress [4,5,55]. Some of these factors might be the target of SchB’s action in *pde6c*^*w59*^ retina. For example, the oxidative stress in the mutant retina [28] might have been reduced by SchB’s antioxidative property [10–14]. Alternatively, SchB might directly inhibit the apoptotic pathway in *pde6c*^*w59*^ rods, or induce a desirable systemic effect that benefited the rods. Nonetheless, the rod cell number was not statistically different between the *pde6c*^*w59*^ mutant and WT at 6 dpf, even though apoptotic rods were detected earlier, at 4 dpf [28]. This is not surprising, given the slow time course of rod death in the *pde6c*^*w59*^ mutants, in which noticeable loss was only apparent in 6-week-old juveniles [22]. Although the SchB treatment benefited *pde6c*^*w59*^ rods, it did not rescue the dying *pde6c*^*w59*^ cones (Fig 4). This differential response of cones to SchB might be caused by their dying through necroptosis [28], a mechanism that might not be a target of SchB. In other words, SchB probably acted on the apoptotic pathway and in turn benefited *pde6c*^*w59*^ rods.

The positive drug effect also enhanced the light sensation of the *pde6c*^*w59*^ mutants, and resulted in their larger Light-On VMR (Fig 3A). This VMR enhancement was substantially mediated by eye function, as it was attenuated by enucleation. This deduction is corroborated by the observation that an eyeless mutant *chokh* did not display a VMR [30] when measured with the same experimental parameters used in this study. These observations therefore strongly implicate that the light perception of *pde6c*^*w59*^ eyes was enhanced by the SchB treatment. Nonetheless, SchB also enhanced the Light-On VMR of the enucleated *pde6c*^*w59*^ larvae starting at 60 s after light onset. This component is therefore likely mediated by extra-ocular PRs, which have been shown to elicit locomotor response towards light offset in a longer time scale [56]. Thus, SchB probably acted on both the eye and extra-ocular levels, which in turn mediated the fast and slow components of the enhanced Light-On VMR respectively in the *pde6c*^*w59*^ mutants.

This eye-level enhancement was likely mediated by the morphologically-improved *pde6c*^*w59*^ rods. This is plausible, as normal rods have been shown to perceive light at this stage of development by two independent investigations. The first one is the detection of rod electroretinogram (ERG) at 5 dpf in a *nof/gnat2* mutant with no cone function [57]. The second is the identification of rod VMR at 6 and 7 dpf in the same *nof/gnat2* mutant [58]. Similar to the *nof/gnat2* rods, *pde6c*^*w59*^ rods were genetically normal and they were dark adapted for 30 minutes before light onset in the VMR assay. Their phototransduction cascade was supposedly intact and should be able to detect light onset, even though these rods were degenerating as the bystanders of cone death. The abnormally-large size of these degenerating rods was also reduced by the SchB treatment. Hence, these observations lead to the hypothesis that the morphologically-improved rods drive the observed VMR enhancement. This hypothesis can be tested by ERG measurement or by ablation of rod function in the *pde6c* ^*w59*^ mutant before VMR assay. The morphological improvement in rods should also be evaluated by electron microcopy to deduce the specific sub-cellular changes after the SchB treatment. The enhanced Light-On VMR, however, was likely not mediated by cones because they were not morphologically improved by the SchB treatment (Fig 4F and Table 3). Furthermore, they carried a permanent mutation in the *pde6c* gene which prohibited normal phototransduction. Thus, it is more likely that SchB acted on rods and in turn enhanced the light sensation of *pde6c*^*w59*^ mutants.

In this study, the positive effect of SchB was first detected by the VMR. This discovery suggests that the VMR can be an *in vivo* assay for evaluating drug effects on visual mutants. Indeed, it has recently been used to evaluate oculotoxic drugs in a study which reported a positive predictive value of 83% [59]. In conjunction with other experimental approaches, the VMR assay will facilitate identification of new drugs for treating retinal degenerative diseases. In this study, it has unveiled a positive effect of SchB on the *pde6c*^*w59*^ retinal-degeneration mutant. This property can potentially be developed to a functional therapy for treating patients who suffered from retinal degeneration with similar pathogenesis.

## Supporting Information

S1 Fig Experimental design for the VMR assay. The VMR-assay design used in this study was adapted from Emran et al., 2008. Before the actual experiment, the 96-well plate with the larvae was placed in the ZebraBox system for 3.5 hours of dark adaption to acclimatize the animals. The data collection was started at 0.5 hours before the first light onset. The actual test consisted of three consecutive trials of light onset (Light-On) and light offset (Light-Off) periods with each period lasted for 30 minutes. The activity of individual larva was extracted from the video data with an approach fully elaborated in the Method section. In short, it is defined as movement duration per second. Finally, the activity of the same type of larvae was averaged across the three ON or OFF trials for plotting, or was used for statistical testing.
(TIF)

S2 Fig Microdissection of larval retina for whole-mount immunostaining. In this study, larval retinas were microdissected from 6-dpf larvae. First, the scleral region just outside the circumference of the pupil was severed from the lateral side of the larvae (black circular arrow in A) by a fine hook created and bent from a chemically-etched tungsten needle [39]. An example of this needle is indicated by the white arrow in (B). In the same figure, the black arrow indicates an insect pin of size 000 (Fine Science Tools, Foster City, CA). Three 6-dpf retinas are shown in the figure to give a reference of the relative size. After severing the scleral attachment from the lateral side (circular arrow in A), the RPE-attached retinas could be easily detached from the sclera by a gentle push from the medial side (A, white arrow). These RPE-retinas were then treated with acetone, which would further detach the RPE from the retinas. The detached RPE was removed from the retinas by the chemically-etched tungsten needle. (C) Finally, the dissected retinas (indicated by the white arrow) were collected in a microcentrifuge tube for downstream immunostaining procedure.
(TIF)

S3 Fig The SchB treatment enhanced the VMR of *pde6c*^*w59*^ larvae through their eyes. This figure shows the same plots as Fig 3, except for the omission of error ribbons to emphasize the activity traces.
(TIFF)

S1 File Raw data files for Fig 2. (ZIP)

S2 File Raw data files for Fig 3. (ZIP)

S3 File Raw data files for Table 2. (ZIP)

S4 File Raw data files for Table 3. (ZIP)
