# Correction: A Naturally-Derived Compound Schisandrin B Enhanced Light Sensation in the *pde6c* Zebrafish Model of Retinal Degeneration

**DOI:** 10.1371/journal.pone.0154552

**Published:** 2016-04-25

**Authors:** Liyun Zhang, Lue Xiang, Yiwen Liu, Prahatha Venkatraman, Leelyn Chong, Jin Cho, Sylvia Bonilla, Zi-Bing Jin, Chi Pui Pang, Kam Ming Ko, Ping Ma, Mingzhi Zhang, Yuk Fai Leung

The affiliation for the eleventh author is incorrect. Ping Ma is affiliated with the Department of Statistics, University of Georgia, 101 Cedar St, Athens, GA 30602, USA.
