# Supplementary figures and images for: A Naturally-Derived Compound Schisandrin B Enhanced Light Sensation in the pde6c Zebrafish Model of Retinal Degeneration
Source: PLoS One. 2016 Mar 1;11(3):e0149663. doi: 10.1371/journal.pone.0149663 (PMC4773124; doi:10.1371/journal.pone.0149663)

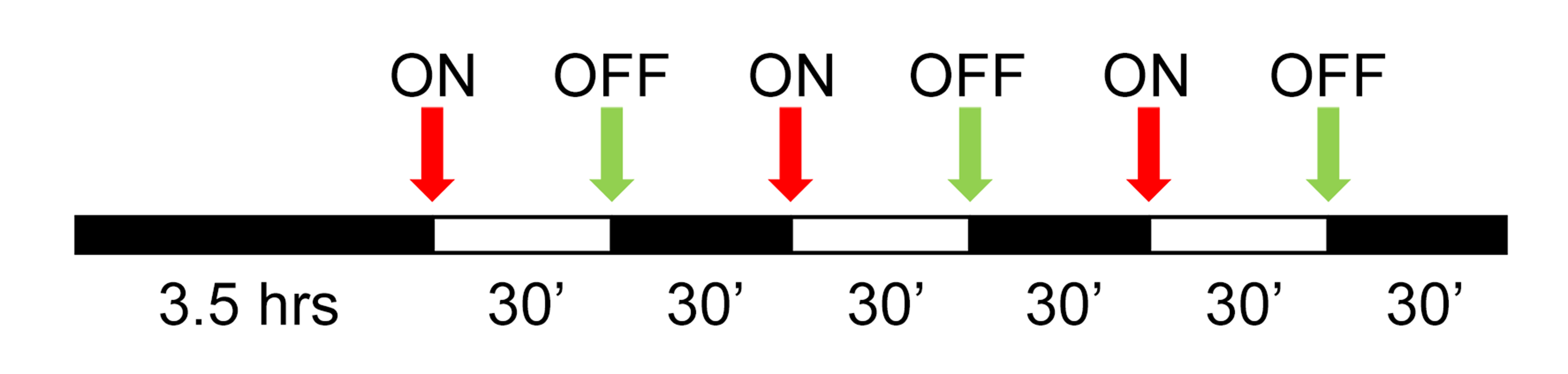

Supplement: S1 Fig — The VMR-assay design used in this study was adapted from Emran et al., 2008. Before the actual experiment, the 96-well plate with the larvae was placed in the ZebraBox system for 3.5 hours of dark adaption to acclimatize the animals. The data collection was started at 0.5 hours before the first light onset. The actual test consisted of three consecutive trials of light onset (Light-On) and light offset (Light-Off) periods with each period lasted for 30 minutes. The activity of individual larva was extracted from the video data with an approach fully elaborated in the Method section. In short, it is defined as movement duration per second. Finally, the activity of the same type of larvae was averaged across the three ON or OFF trials for plotting, or was used for statistical testing. (TIF) [file pone.0149663.s001.tif]

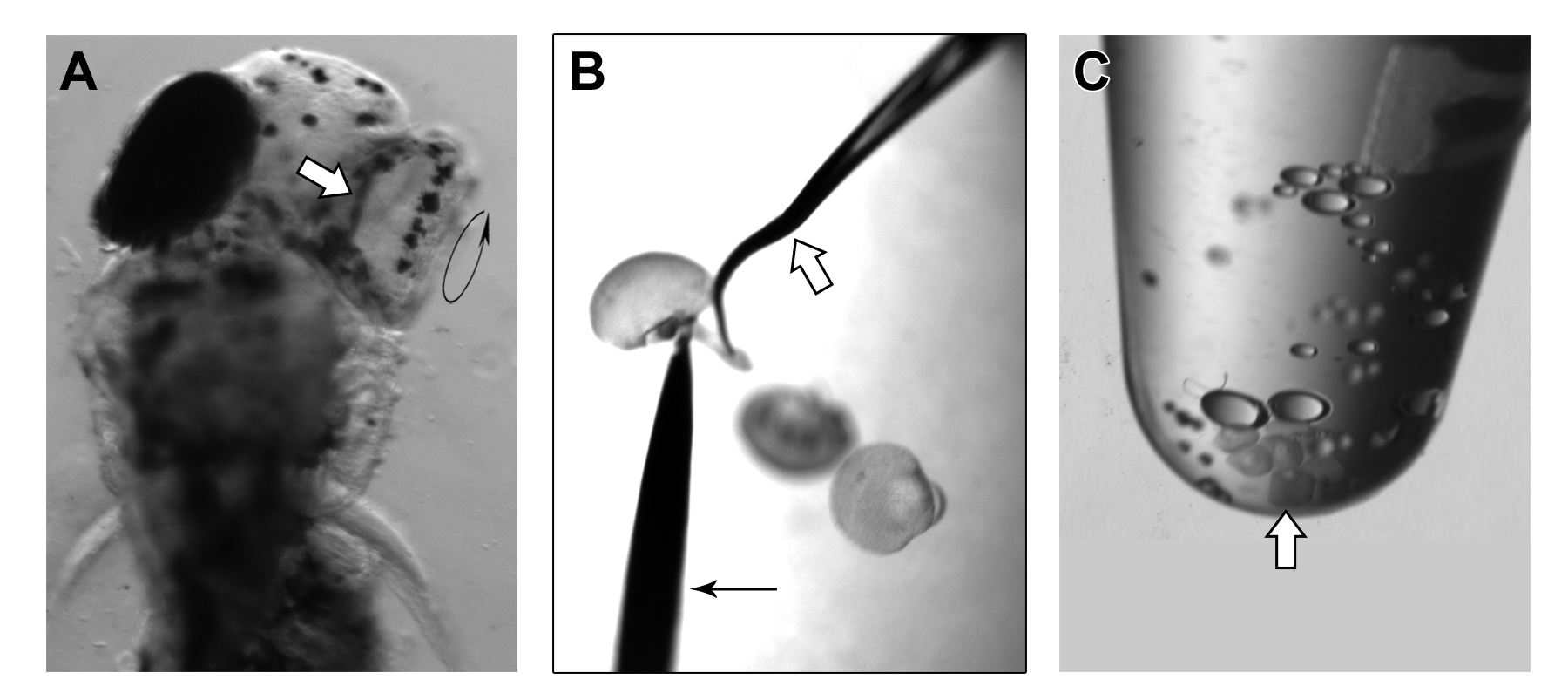

Supplement: S2 Fig — In this study, larval retinas were microdissected from 6-dpf larvae. First, the scleral region just outside the circumference of the pupil was severed from the lateral side of the larvae (black circular arrow in A) by a fine hook created and bent from a chemically-etched tungsten needle [39]. An example of this needle is indicated by the white arrow in (B). In the same figure, the black arrow indicates an insect pin of size 000 (Fine Science Tools, Foster City, CA). Three 6-dpf retinas are shown in the figure to give a reference of the relative size. After severing the scleral attachment from the lateral side (circular arrow in A), the RPE-attached retinas could be easily detached from the sclera by a gentle push from the medial side (A, white arrow). These RPE-retinas were then treated with acetone, which would further detach the RPE from the retinas. The detached RPE was removed from the retinas by the chemically-etched tungsten needle. (C) Finally, the dissected retinas (indicated by the white arrow) were collected in a microcentrifuge tube for downstream immunostaining procedure. (TIF) [file pone.0149663.s002.tif]

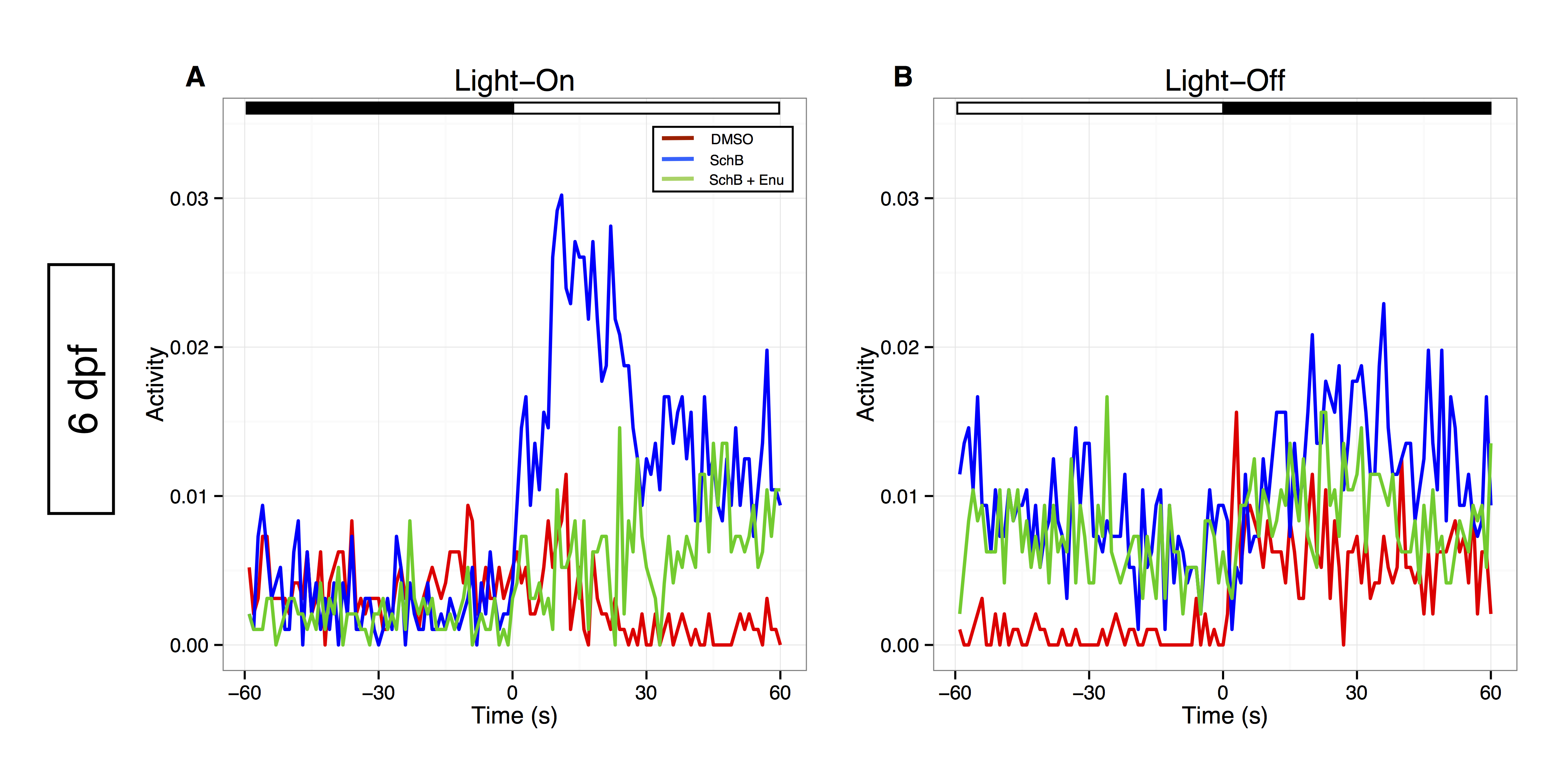

Supplement: S3 Fig — This figure shows the same plots as Fig 3, except for the omission of error ribbons to emphasize the activity traces. (TIFF) [file pone.0149663.s003.tiff]
